# The Care of Poisons

**Published:** 1907-04-06

**Authors:** 


### THE CARE OF POISONS.

BY A LONDON MATRON.

Few matters under the matron's control are of more vital importance than the safe keeping and proper administration of poisons. Apart from the grave issues which depend upon the maintenance of proper regulations for safeguarding poisons in the hospital, it is the method learned while a probationer that the private nurse will adopt with her patients, and thus it is imperative that a full sense of responsibility should early be taught. It is in the hands of the ward sister that responsibility as to the careful control and administration of poisons must chiefly lie, and it is her duty to be incessantly on her guard lest familiarity render her subordinates forgetful of the risks involved in the careless handling of medicines and poisons.

Poisons in a ward are divided into "lotions," and the contents of the "poison cupboards."

Lotions, as a rule, are kept in jars on a stand or dressing-trolley well away from the patients' beds. They are usually well diluted and coloured. Thus, when lotion-jars are distinctly marked all mistakes with regard to lotions can be avoided. With the exception of these coloured and diluted poisonous lotions, all poisons are kept under lock and key in the familiar

"Poison Cupboard,"

which should always be carefully kept locked and the key deposited in some recognised place in the ward. The ward sister, or staff nurse, should alone have access to the poison cupboard. This is a very important rule, as it impresses young nurses with the responsibility attached to the use of poisons, and ensures that the more experienced worker has this important duty entirely in her hands.

The contents of a poison cupboard are, in the first place, a certain number of "stock" poisons, chiefly narcotics, which vary in different hospitals, and of which a printed list should be kept in each cupboard; those of a highly poisonous nature should be in blue or green ribbed bottles, while others such as pot. brom. are generally in white bottles. In the second place come all liniments or medicines of a poisonous character ordered for individual patients. These should invariably be kept in the poison cupboard in coloured bottles, and it is a good practical plan to keep there also a small tray with hypodermic syringes and drugs likely to be needed in an emergency ready for immediate use.

In a hospital with several wards the contents of all poison cupboards with regard to "stock" poisons should be identical and all bottles of different sizes and colours should be used for the same drugs throughout the hospital, with the name and strength of the poison printed, or legibly written, on each bottle. It is advisable that from time to time a visit of inspection should be paid to the ward poison-cupboards by the medical superintendent, head dispenser, or an official deputed to do so; this acts as a check on those disposed to overstock the cupboards with drugs "that may be ordered some day," and also prevents drugs whose therapeutic values depreciate in time from being used when inefficacious.

It is a good rule that no poison-cupboard should contain other than recognised "stock" drugs and those that may be in use for a patient actually in the ward.

### Private Practice.

The nurse in private has more difficulty, and in many ways more anxiety, with regard to poisons than the nurse working in hospital, and she should be careful to carry out the principles, and, if possible, the practice taught her in her training school.

All bottles marked "Poison" should be under lock and key, and lotions should be coloured. A bowl containing lotion should never be carelessly put down, especial precautions being taken in a house where there are children.

Many unfortunate, even fatal mistakes are made owing to want of care and lack of common sense with regard to poisons in private houses, which might have been obviated had due responsibility in this respect been learnt in the wards.

### Witnessing of Poisons.

No poison, and, it may be added, no sleeping-draught, should be administered by a nurse unless it is witnessed by someone competent to do so. This rule applies particularly to drugs and draughts given from a "stock" bottle. In a hospital the witnessing is usually done on day duty by a sister or staff nurse, and on night duty by the night sister. Too much importance cannot be attached to this point, as an efficient witness acts as a safeguard to the patient and also to the nurse herself.

The order for the injection or draught signed by the surgeon or physician should be shown; the bottle from which the dose has been seen to be taken should be verified, and the amount carefully noted. All these details should be *verbally* repeated and a perfunctory "Yes" should not be accepted as a sufficient guarantee that all is correct.

In conclusion, those who are training nurses cannot too strongly recognise the importance of impressing the probationer with the grave dangers attached to all work in connection with lotions, medicines, and poisons. In order to develop a sense of responsibility in each nurse, she should, early in her career, be instructed in the use and preparation of lotions, and be unceasingly reminded that nothing should be taken from a bottle or lotion jar without looking at the label, and that all amounts should be carefully *measured*, not guessed. A busy ward sister will find that a little time thus spent with the "new nurse" will amply repay her for the time and trouble; and good habits can thus be inculcated early in a nurse's career that will render her less likely to make mistakes when, as a senior nurse, the responsibility of administering drugs falls to her lot.

Later on in her career a nurse should be taught to give medicines, etc., under the immediate supervision of the sister, and she thus becomes gradually fitted to be left in charge of a ward with all the grave responsibility of giving many and various drugs to patients whose lives are in this way literally in her hands.

